# Mutation-Tolerant Inhibition of HIV-1 Integrase Strand Transfer by Secondary Metabolites from the Endophytic Fungus *Alternaria alternata* PO4PR2

**DOI:** 10.3390/microorganisms14051102

**Published:** 2026-05-13

**Authors:** Ndzalo Mashabela, Darian Naidu, Ernest Oduro-Kwateng, Nompumelelo P. Mkhwanazi

**Affiliations:** HIV Pathogenesis Programme, School of Medicine, College of Health Science, University of KwaZulu-Natal, Durban 4000, South Africa; 223023260@stu.ukzn.ac.za (N.M.); 218052764@stu.ukzn.ac.za (D.N.); 223149553@stu.ukzn.ac.za (E.O.-K.)

**Keywords:** endophytic fungi, HIV-1 antiviral activity, INSTI drug-resistance mutation, coumarin derivatives, molecular docking, mutation-tolerant inhibition

## Abstract

Endophytic fungi are promising sources of novel antiviral compounds, and the crude extract from *Alternaria alternata* PO4PR2 has previously shown anti-HIV-1 activity. This study evaluated its efficacy against integrase strand-transfer inhibitor (INSTI)-resistant HIV-1 and its mechanism of action. Key resistance mutations (Y143H, G118R, N155H, and R263K) were introduced into the HIV-1 pNL4.3 clone via site-directed mutagenesis and confirmed through Sanger sequencing. Viral infectivity was assessed in TZM-bl cells, while cytotoxicity was measured using an MTT assay. Antiviral activity was determined through a luciferase-based assay, and integration inhibition was evaluated using integrase activity assays and *Alu-gag* nested PCR. The extract demonstrated potent inhibition of resistant mutants, with low IC_50_ values (0.02971–0.1652 μg/mL), and showed minimal cytotoxicity (CC_50_ = 300 μg/mL), maintaining over 80% cell viability. It inhibited integrase activity by 67%, specifically targeting the strand-transfer step, and significantly reduced integrated viral DNA. Molecular docking of 14 compounds identified coumarin derivatives as key bioactive metabolites, exhibiting mutation-tolerant binding within the integrase catalytic pocket. Overall, these findings highlight PO4PR2 as a promising source of compounds for developing new therapies targeting drug-resistant HIV-1 integrase.

## 1. Introduction

Although major strides have been made in the management of human immunodeficiency virus (HIV) infection, the virus continues to pose significant public health challenges, affecting 40.8 million people globally as of 2025 [1]. The burden of HIV remains disproportionately high in sub-Saharan Africa, with Southern Africa bearing the greatest share of the global epidemic. In South Africa alone, an estimated 7.6 million people are currently living with HIV. While antiretroviral therapy (ART) has transformed HIV into a manageable chronic condition, the inability to eliminate latent reservoirs [2,3], persistent drug toxicities, and the emergence of drug resistance underscore the urgent need for novel therapeutic strategies [4,5,6,7].

HIV-1 integration is a critical and irreversible step in the viral replication cycle catalyzed by the integrase (IN) enzyme. This process involves two distinct stages: 3′ processing, which prepares the viral DNA ends, and strand transfer, which covalently inserts the viral DNA into the host genome [8,9,10,11]. Most current integrase strand-transfer inhibitors (INSTI), such as Raltegravir and Dolutegravir, target the latter. However, specific mutations in the catalytic pocket can hinder drug binding, leading to virological failure [12,13]. Identifying bioactive compounds that maintain efficacy against these drug-resistant strains is therefore a high priority for next-generation drug development.

In the search for alternative scaffolds, endophytic fungi have emerged as promising sources of novel bioactive metabolites [14,15]. Members of the genus *Alternaria* spp. have demonstrated significant inhibitory activity against HIV [16,17,18]. For instance, altertoxins isolated from *Alternaria tenuissima* QUE1Se demonstrated complete inhibition of HIV-1 replication in T-cells at low micromolar concentrations, while other species have exhibited strong inhibitory effects against reverse transcriptase and protease [18,19]. Previous studies of the endophytic fungus *Alternaria alternata* P04PR2 established its potent broad spectrum anti-HIV activity across diverse cell lines and HIV-1 subtypes [20,21]. Subsequent results identified several secondary metabolites, including benzofuran and coumarin derivatives, as potential drivers of this activity [22]. However, a critical knowledge gap remains, and the specific functional efficacy of these metabolites against major INSTI resistance pathways (G118R, N155H, R263K, and Y143R) has not been quantified. Furthermore, the molecular basis for the extract’s continued efficacy against variants that compromise second-generation inhibitors, like Dolutegravir, remains elusive.

This study addresses these gaps by providing a quantitative assessment of the inhibitory mechanism of *A. alternata* PO4PR2 extract. By integrating site-directed mutagenesis, *Alu-gag* nested PCR, and molecular docking, we delineate the site-specific (N155H, Y143R, G118R, and R263K) interaction between these fungal metabolites and mutated integrase enzyme. This research provides the functional evidence necessary to transition these natural products from crude extracts to targeted, mutation-tolerant therapeutic leads.

## 2. Materials and Methods

### 2.1. Selection of Drug-Resistance Mutations (DRMs)

To identify integrase mutations, 122 integrase sequences were retrieved from the Stanford HIV Drug Resistance and Los Alamos HIV Sequence Databases using HXB2 as a reference sequence. This dataset was supplemented with Southern Africa HIV-1 subtype C sequences from published datasets [23,24,25], resulting in a composite dataset of 156 sequences (Accession Nos: MW690052-MW690089, MG989443.1, MG989444.1, MW328641-713, and MK899435-MK899477). Resistance levels were determined using Stanford HIVDB Program cumulative penalty scores: susceptible (Score 0–9), low-level resistance (15–29), intermediate resistance (30–59), and high-level resistance (≥60). Based on these profiles, G118R, N155H, R263K, and Y143R were selected for functional validation due their impact on second-generation INSTIs (Table 1).

### 2.2. Isolation and Preparation of A. alternata PO4PR2 Extract

The endophytic fungus *A. alternata* P04PR2 was previously isolated from *Hypoxis* spp. and *S. birrea* and cultured in potato dextrose broth (PDB) for 14 days at 25 °C. Secondary metabolites were extracted using absolute methanol (1:1 *v*/*v*) under agitation (150 rpm) for 24 h. The supernatant was filtered, evaporated to dryness at 40 °C, and stored at −80 °C. The crude extract was reconstituted in distilled water to a stock concentration of 400 μg/mL [20].

### 2.3. Cell Culture Maintenance

TZM-bl cells (HeLa modified to express CD4, CCR5, and CXCR4 with HIV-1 LTR-driven luciferase and β-galactosidase reporters) and HEK 293T cells were maintained in DMEM (Thermo Fisher Scientific, Waltham, MA, USA) supplemented with 10% heat-inactivated FBS, 25 mM HEPES, and 50 µL/mL gentamicin. Cultures were incubated at 37 °C in 5% CO_2_. Cell viability and density were monitored using the Trypan Blue exclusion method and a hemocytometer to ensure confluency prior to trypsinization with 0.25% Trypsin-EDTA [26,27].

### 2.4. Site-Directed Mutagenesis

Mutagenic primers (Table 2) were designed using the NEBaseChanger primer design tool (New England Biolabs, MA, USA). Mutations were introduced into the HIV-1 molecular clone pNL4.3 using the Q5 site-directed mutagenesis kit (New England Biolabs, Ipswich, MA, USA). PCR reactions (25 µL) utilized the Q5 Hot start High-fidelity Master Mix and 1–25 ng of template DNA. Thermocycling included an initial denaturation at 98 °C for 30 s; 25 cycles of 98 °C for 10 s, 55–60 °C for 20 s (gradient), and 72 °C for 100 s; with a final extension at 72 °C for 2 min. Amplified PCR products were treated with KLD enzyme mix (Kinase, Ligase, DpnI) for 5 min at 25 °C to eliminate parental methylated DNA.

#### Transformation of Competent *Escherichia coli* Cells

KLD-treated DNA (5 µL) was transformed into chemically competent *Escherichia coli* (*E. coli*) via heat shock at 42 °C for 30 s. Following outgrowth in SOC medium at 37 °C for 1 h, cells were plated on LB agar containing 100 mg/L ampicillin. Positive colonies were expanded in LB broth, and plasmid DNA was extracted using the Gene jet Plasmid Miniprep Kit (Thermo Fisher). The quality and purity of the extracted plasmid DNA were assessed using 1% gel electrophoresis and Nanodrop quantification.

### 2.5. Sequence Validation of Integrase Mutations

#### 2.5.1. PCR Amplification of the Integrase Gene

The integrase gene of the selected mutants was amplified using the HotStarTaq Master Mix (QIAGEN, Hulsterweg, The Netherlands). Each 50 µL reaction contained 25 μL of Master mix, 1 μL each of primers 4155F-(5′-GTACCAGCACACAAAGGRATTG-3′) and 5264R-(5′-CCTGTATGCAGACCCCAATATGTT-3′), and 1 μL of DNA template. Thermal cycling was performed on a MiniAmp thermal cycler (Thermo Fisher Scientific, Waltham, MA, USA) under the following conditions: 1 cycle at 95 °C for 5 min, 1 cycle at 94 °C for 1 min, then 55 °C for 1 min, then 72 °C for 3 min and 30 s for 30 cycles, followed by final extension at 72 °C for 10 min. Amplicons were verified via Nanodrop quantification and 1% gel electrophoresis.

#### 2.5.2. Cycle Sequencing Reaction

Sequencing reactions were prepare using the BigDye Terminator v3.1 cycle sequencing kit (Thermo Fisher Scientific, Waltham, MA, USA). Reactions (10 µL) consisted of 2 µL of Sequencing Buffer, 3.2 μL of 10 μM forward and reverse primers (4155F-GTACCAGCACACAAAGGRATTG and 5264R-CCTGTATGCAGACCCCAATATGTT), 0.4 μL of BigDye Terminator v3.1 Ready Reaction Mix, and 20 ng of DNA template. M13-TGTAAAACGACGGCCAGT and pGEM were used as positive controls for reagent validation. The cycling conditions were 96 °C (1 min), 96 °C (10 s), 50 °C (5 s), and then 60 °C (4 min) for 25 cycles.

#### 2.5.3. Purification and Capillary Electrophoresis

Sequencing products were purified using EDTA/ethanol precipitation. Briefly, 1 µL of 125 mM EDTA (pH 8.0) and 26 μL of pre-mix of NaOAc (pH 5.2) solution were added per well, followed by centrifugation at 3000× *g* for 20 min. Pellets were washed with 35 μL of 70% cold ethanol, dried at 50 °C, and resuspended for analysis using the 3500 genetic Analyzer (Applied Biosystems, Waltham, MA, USA). Full-length integrase sequence was assembled using Contig Assembly Tools v3 and aligned in Unipro UGENE (version 50.0, Unipro) to confirm the presence of the targeted mutations.

#### 2.5.4. Plasmid DNA Preparation and Purification

Once confirmed, 100 µL of glycerol stocks of the G118R, N155H, R263K, and Y143R plasmids were inoculated into 100 mL of prewarmed LB (Luria–Bertani) broth supplemented with 100 µL of 1% ampicillin and shaken overnight at 37 °C in a shaking incubator at 230 rpm. High-yield plasmid DNA was purified using the QIAGEN Plasmid Maxi Kit (QIAGEN, Hulsterweg, The Netherlands) in accordance with the manufacturer’s guidelines. DNA quality (A_260_/A_280_ ≈ 1.8) was verified via Nanodrop One^TM^ quantification and 1% gel electrophoresis prior to transfection.

### 2.6. Cytotoxicity and Cell Viability Assay

Cytotoxicity was evaluated using the CyQUANT MTT Cell Proliferation Kit (Thermo Fisher, Waltham, USA) [22]. TZM-bl cells (15,000/well) were seeded in 96-well plates and treated with serial dilutions of *A. alternata* P04PR2 extract or Azidothymidine (AZT) as a control. Following 48 h of incubation, 10 μL of 12 mM MTT solution was added for 4 h, followed by solubilization in 50 μL DMSO. Absorbance was measured at 540 nm. The 50% cytotoxic concentration (CC_50_) was determined via non-linear regression using GraphPad Prism 5 based on the following formula:

$$\%Cell\;Viability=(\frac{Sample\;A\;bsorbance-blank}{Mean\;media\;control\;absorbance-blank})\times 100$$

### 2.7. Generation of HIV-1 Mutant Stocks

Mutant HIV-1 stocks were generated by transfecting 3 × 10^6^ HEK293T cells in T-75 flasks. Cells were transfected with 12 µg of mutated pNL4.3 plasmid DNA using FuGENE^®^ HD Transfection Reagent (Promega, Wisconsin, WI, USA) according to the manufacturer’s instructions. Supernatants were harvested 48 h post-transfection, filtered through a 0.45 μm membrane, and stored at −80 °C. Viral infectivity was quantified via TCID_50_ titration on TZM-bl cells.

### 2.8. Viral Titration (TCID_50_)

Viral titres were determined in TZM-bl cells via a single-cycle infection assay. Briefly, virus stocks were serially diluted in a 96-well cell culture plate before adding 10^4^ TZM-bl cells supplemented with 37.5 μg/mL DEAE-dextran (diethylaminoethyl-dextran). After 48 h of incubation at 37 °C (5% CO_2_), 100 μL of cell culture media was extracted and 100 μL of Bright Glo^TM^ luciferase reagent (Promega) was added to each well to induce cell lysis. Luminescence was measured as relative light units (RLU) using a Victor Nivo microplate reader (PerkinElmer, Waltham, MA, USA). The TCID_50_ was defined as the dilution producing between 15,000 and a maximum of 50,000 RLUs.

### 2.9. Luciferase-Based Antiviral Assay

The inhibitory activity of the extract against G118R, N155H, R263K, and Y143R mutants was tested using a luciferase reporter assay, as previously described [20,21,22]. Briefly, 96-well culture plates (Corning, NY, USA) were prepared by adding 150 μL, 100 μL, and 140 μL of supplemented DMEM (10% FBS, 25 mM HEPES buffer and 1% penicillin–streptomycin) to wells designated as cell controls, virus control, and test samples, respectively. Subsequently, 11 μL of the drug controls (Azidothymidine, Raltegravir, and Dolutegravir) and *A. alternata* PO4PR2 crude extract were added, followed by serial 3-fold dilution. The cells were then infected with 50 μL of the respective viruses at a calculated TCID_50_ and incubated for 1 h. Following infection, 10^4^ TZM-bl cells per well, supplemented with 37.5 μg/mL DEAE-dextran, were added to the culture plate. The culture was maintained for 48 h at 37 °C, 5% CO_2_, and 95% humidity.

Post-incubation, 150 μL of medium was decanted and replaced with 100 μL of Bright Glo luciferase reagent (Promega, Madison, WI, USA) in the absence of light. The luminance was immediately measured using the Victor Nivo microplate reader at 540 nm (PerkinElmer, Waltham, USA). All experiments were independently repeated three times, and results are expressed as the mean ± standard deviation (SD). The percentage of viral inhibition was calculated relative to the uninfected cell control and infected viral control using the following formula:

$$\%HIV\;inhibition=[1-(\frac{sample\;RLU-cell\;control\;RLU}{viral\;control\;RLU-cell\;control\;RLU})]\times 100$$

A dose–response curve was used to calculate the IC_50_ (inhibitory concentration) using GraphPad Prism software (Version 5).

### 2.10. HIV Integrase Assay

Biochemical inhibition of HIV-1 integrase was quantified using the HIV Integrase Assay Kit EZ-1700 (Express Biotech International, Fredrick, MD, USA) [28]. Briefly, 100 μL of 1x double-stranded (DS) DNA was immobilized on streptavidin-coated 96-well plates for 30 min at 37 °C. Wells were washed five times with 300 μL of wash buffer and blocked for 30 min. Following blocking, 100 μL of recombinant HIV-1 integrase enzyme was added and incubated for 30 min at 37 °C. To evaluate inhibition, 50 μL of *A. alternata* PO4PR2 crude extract, Raltegravir (positive control), or reaction buffer (negative control) was added to the respective wells and pre-incubated for 5 min at room temperature. The strand-transfer reaction was initiated by the addition of 50 μL 1X target substrate (TS) DNA and incubated for 30 min at 37 °C. Following a final wash cycle, 100 μL of HRP-conjugated antibody was added (30 min, 37 °C). The reaction was developed using TMB peroxidase substrate and terminated with 100 μL of TMB stop solution. Absorbance was measured at 450 nm using the Victor Nivo microplate reader (PerkinElmer, Waltham, MA, USA). All experiments were independently repeated three times, and results are expressed as the mean ± standard deviation (SD).

### 2.11. Quantification of Integrated HIV-1 DNA via Alu-Gag Nested PCR

Peripheral blood mononuclear cells (PBMCs) from HIV-1-uninfected participants were sourced from the Female Rising through Education, Support and Health (FRESH) cohort, an ongoing prospective study in KwaZulu-Natal, South Africa [29,30]. Cryopreserved PBMCs were thawed rapidly at 37 °C and transferred into 8 mL of pre-warmed R10 medium (RPMI-1640, Gibco, supplemented with 10% FBS, 1% L-glutamine, 1% penicillin-streptomycin, and 15 HEPES). Cells were centrifuged at 1500 rpm for 10 min at room temperature, washed in fresh R10, and allowed to rest for 2 h at 37 °C in a 5% CO_2_ incubator.

For experimental treatments, 1 × 10^5^ PBMCs per well were seeded in 24-well plates. Cells were treated with 20 µL of either *A. alternata PO4PR*2 crude extract or DTG as a positive antiviral control, while untreated PBMCs served as a negative control. Cells were infected with 100 µL of mutant HIV-1 (at calculated TCID50) and incubated for three days. Genomic DNA (gDNA) was subsequently isolated from the PBMCs using the QIAamp DNA Blood Mini Kit (QIAGEN, Hilden, Germany), and DNA concentration and purity were assessed via Nanodrop One quantification (Thermo Fisher Scientific).

Integration was quantified using a two-step nested *Alu-gag* PCR. The first round amplified the junction between the host Alu elements and the viral gag gene. Reactions (25 µL) contained 1x PCR buffer, 1.5 mM MgSO_4_, 0.5 mM dNTPs, 1 μM Alu-forward primer, 6 μM Gag-reverse primer, and 150 ng of gDNA. The second round (nested qPCR) targeted the HIV-1 LTR/gag region using 2 μL of the first-round product, *Alu-Gag*-specific primers, and a 6FAM-labeled probe. Human GAPDH (or RNase P) was amplified in parallel to normalize for cellular DNA input.

*Alu-gag* PCR was performed as follows: The first-round PCR reaction was prepared on ice using 1X PCR buffer (Thermo Fisher), 1.5 mM MgSO4 (Thermo Fisher), 0.5 mM dNTPs (Thermo Fisher), 1 μM *Alu* forward primer (5′-GCC TCC CAA AGT GCT GGG ATT ACA G-3′), 6 μM *Gag* reverse primer (5′-TCG CTT TCA GGT CCC TGT TCG-3′), 2.5 U Platinum Taq polymerase (Thermo Fisher), and 150 ng of genomic DNA. The cycling conditions were as follows: initial denaturation of 95 °C for 2 min, followed by 14 cycles of denaturation at 95 °C for 30 s and annealing at 50 °C for 30 s, and extension at 72 °C for 210 s. The nested PCR reaction was then prepared on ice using 1X PCR buffer (Thermo Fisher), 1.5 mM MgSO4 (Thermo Fisher), 0.5 mM dNTPs (Thermo Fisher), 0.5 μM *AluGag* forward primer (5′-GGT GCG AGA GCG TCA GTA T-3′) and 0.5 μM *AluGag* reverse primer (5′-AGC TCC CTG CTT GCC CAT A-3′), 0.15 μM *AluGag* probe (6FAM-AAA ATT CGG TTA AGG CCA GGG GGA AAG AA-QSY7), 1 U Platinum Taq polymerase (Thermo Fisher), and 2 μL of *Alu-gag* PCR product. The cycling conditions were as follows: 95 °C for 5 min, followed by 50 cycles of 95 °C for 10 s and 60 °C for 30 s. Human GAPDH was used for normalization (Thermo Fisher). The experiment was performed in three independent replicates.

### 2.12. Computational Systems Preparation

The high-resolution structure of the wild-type HIV-1 integrase (IN) intasome in complex with viral DNA, two Mg^2+^ ions, and Dolutegravir (PDB ID: 8FN7) was retrieved from the RCSB Protein Data Bank (https://www.rcsb.org/) [31]. Missing residues were modeled using MODELLER 10.6, and the best model was selected based on the lowest discrete optimized protein energy (zDOPE) score [32]. Drug-resistant variants (G118R, N155H, Y143R, and R263K) were generated from the wild-type template using the Dunbrack Rotamer Library 2010, implemented in UCSF Chimera 1.19 [33]. All systems were prepared by retaining the viral DNA and Mg^2+^ ions, as these cofactors are essential for maintaining the active site conformation of IN [34]. Protein–DNA complexes were prepared by removing crystallographic water molecules, adding polar hydrogens, and assigning atomic charges using the AMBER force field [35].

The three-dimensional (3D) structures of 14 secondary metabolites from *Alternaria alternata* PO4PR2 (previously characterized experimentally) [22] and the reference inhibitors Dolutegravir (DTG) and Raltegravir (RAL) were obtained from PubChem (https://pubchem.ncbi.nlm.nih.gov/) [36]. Ligands were geometrically optimized in Avogadro 1.2.0 using the generalized AMBER force field (GAFF), followed by final energy minimization in UCSF Chimera 1.19, which employed 100 steps of steepest descent and 10 steps of conjugate-gradient minimization. Hydrogens were added, Gasteiger charges were assigned, and all prepared structures were saved in PDBQT format for docking [35].

### 2.13. Molecular Docking Calculations and Analysis

Molecular docking calculations were performed using Auto Dock Vina 1.1.2 [37]. The grid box was centered within the IN catalytic core–DNA interface at coordinates (x = 55.13, y = 72.57, z = 83.56) with dimensions (x = 24.53 Å, y = 18.71 Å, z = 22.83 Å). The docking parameters were set as follows: number of modes = 10, exhaustiveness = 16, and energy range = 3 kcal/mol. All ligands were docked against the wild-type and mutant HIV-1 IN–DNA–Mg^2+^ complexes. The lowest energy poses were selected based on the binding affinity (kcal/mol) and inspected for key interactions with the catalytic residues (ASP64, ASP116, and GLU152), Mg^2+^ ions, and DNA bases. Protein–ligand complexes were analyzed using BIOVIA Discovery Studio Visualizer 2024 (V24.1.0.23298), highlighting hydrogen bonds, hydrophobic contacts, van der Waals interactions, and metal coordination interactions [22]. Comparative interaction profiling between the reference inhibitors and *A. alternata* PO4PR2 metabolites provided structural insights into their potential mechanisms of inhibition of HIV-1 IN.

### 2.14. Statistical Analysis

All experiments were performed in triplicate across at least three independent trials, and results are expressed as the mean ± standard deviation (SD). Concentration–response data were analyzed using non-linear regression (four-parameter logistic curve) in GraphPad Prism (Version 5) to calculate the IC_50_ and CC_50_ values. Statistical significance between the wild-type and mutant strains was determined using one-way ANOVA followed by Tukey’s post hoc test for multiple comparisons. A *p*-value < 0.05 was considered statistically significant.

## 3. Results

This study evaluated the anti-HIV activity of *A. alternata* PO4PR2 crude extract against a panel of HIV-1 integrase strand-transfer inhibitor (INSTI)-resistant variants. Major INSTI-resistance mutations (G118R, N155H, R263K, and Y143R) were identified through the Stanford Drug Resistance database and introduced into the pNL4-3 plasmid using site-directed mutagenesis. Successful introduction of these substitutions was confirmed through Sanger sequencing and subsequent bioinformatic alignment.

### 3.1. Identification and Selection of HIV-1 Integrase Drug-Resistant Mutants

**Figure 1:**
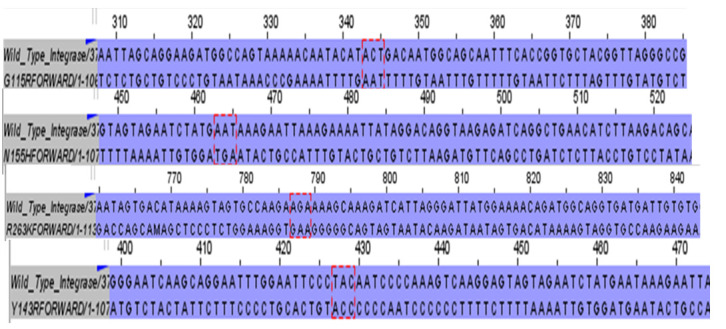
Sequence alignment of HIV-1 integrase mutants. The figure displays the alignment of the site-directed mutants against the wild-type integrase gene, confirming the successful introduction of the G118R, N155H, R263K, and Y143R substitutions.

Drug-resistant HIV-1 integrase mutations were prioritized based on their clinical prevalence and documented contribution to resistance against first- and second-generation INSTIs, including Raltegravir, Elvitegrair, and Dolutegravir. The four selected mutations (G118R, N155H, R263K, and Y143R) represent distinct resistance pathways to verify the accuracy of the site-directed mutagenesis. Full-length integrase sequences were assembled and aligned against the wild-type pNl4.3 refrence using BioEdit (version 7.7.1) and Jalview (version 2.11.5.0) software (Figure 1).

### 3.2. Cytotoxicity and Cell Viability of Alternaria alternata PO4PR2 Crude Extract

**Figure 2:**
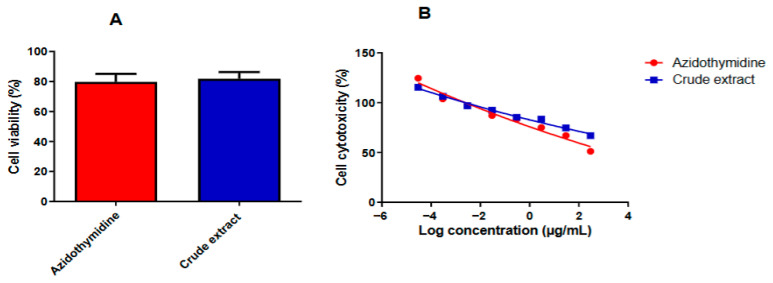
**Cell viability and cytotoxicity (CC_50_) of *A. alternata* PO4PR2 crude extract**. (**A**) Percent cell viability of TZM-bl cells following treatment. (**B**) Dose–response curves used for CC_50_ determination. The blue line represents the crude extract (CC_50_ = 300 µg/mL), and the red line represents Azidothymidine (CC_50_ = 385 µg/mL). Values are expressed as the mean ± standard deviation (SD) of three independent experiments performed in triplicate.

The cytotoxicity of the *A. alternata* PO4PR2 crude extract and drug control, Azidothymidine (AZT), was evaluted in TZM-bl cells using the MTT assay. As shown in Figure 2A, the extract maintained cell viability above 80% across the tested concentrations, indicating low host cell toxicity.

Non-linear regression analysis of the dose–response curves (Figure 2B) determined the CC_50_ for the crude extract to be 300 µg/mL, while AZT exhibited a CC_50_ of 385 μg/mL. The x-axis represents log-transformed concentrations ranging from 300 to 0.00003 μg/mL (log 2.47 to −4.52). These results confirm that the *A. alternata* PO4PR2 extract is non-toxic to TZM-bl cells and possesses a safety profile comparable to that of the reference drug, AZT, within the experimental range.

#### 3.2.1. Molecular Validation and Infectivity of HIV-1 Integrase Mutants

Site-directed mutagenesis was successfully employed to introduce five primary INSTI-resistance mutations (G118R, N155H, R263K, Q148R, and Y143R) into the pNL4.3 molecular clone. Sanger sequencing of the full-length integrase gene confirmed the targeted nucleotide substitutions (e.g., GGA → CGA for G118R); AAC → CAT for N155H) with no adventitious mutations observed in the surroundings regions (Figure 1).

**Figure 3:**
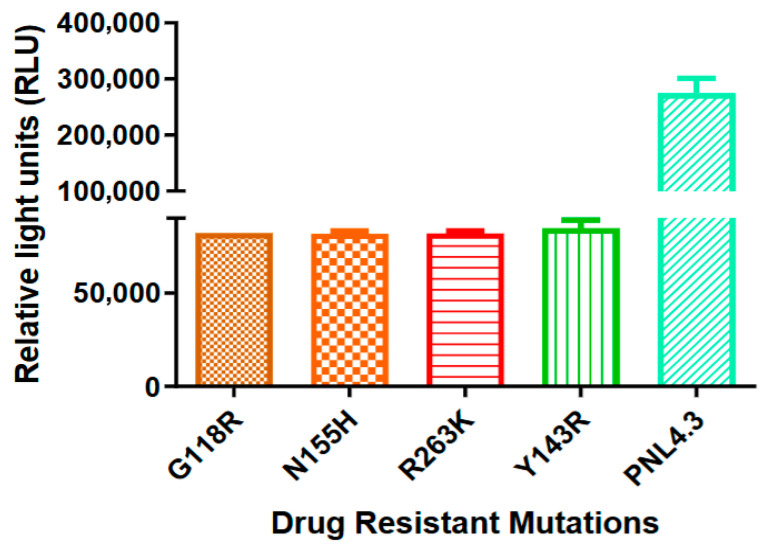
**Comparative infectivity of HIV-1 integrase mutants.** Viral fitness was assessed by measuring relative light units (RLU) in TZM-bl cells. All site-directed mutants produced RLUs well above the 15,000-infectivity threshold, although they exhibited reduced replication efficiency relative to the wild-type pNL4.3 virus. Data are presented as the mean ± SD of three independent experiments.

Following transfection of HEK293T cells, infectious viral stocks were harvested and titrated on TZM-bl cells to determine the 50% tissue culture infection dose (TCID_50_). A threshold of 15,000 relative light units (RLU) was established as the minimum requirement for a strain to be deemed replication-competent for downstream assays. All mutant variants exceeded this benchmark, producing RLU valued above 80,000 RLU (Figure 3).

While the mutants maintained sufficient replicative capacity for antiviral testing, the wild-type pNL4.3 virus generated significantly higher luminescence (270,000 RLU), reflecting the expected fitness cost associated with resistance-associated substitutions. These results indicate that while G118R, N155H, R263K, and Y143R variants remain highly infectious in TZM-bl cells, their replication efficiency is reduced compared to the wild-type reference.

#### 3.2.2. Antiviral Activity of *Alternaria alternata* PO4PR2 Crude Extract Against Integrase Drug-Resistant Mutants

**Figure 4:**
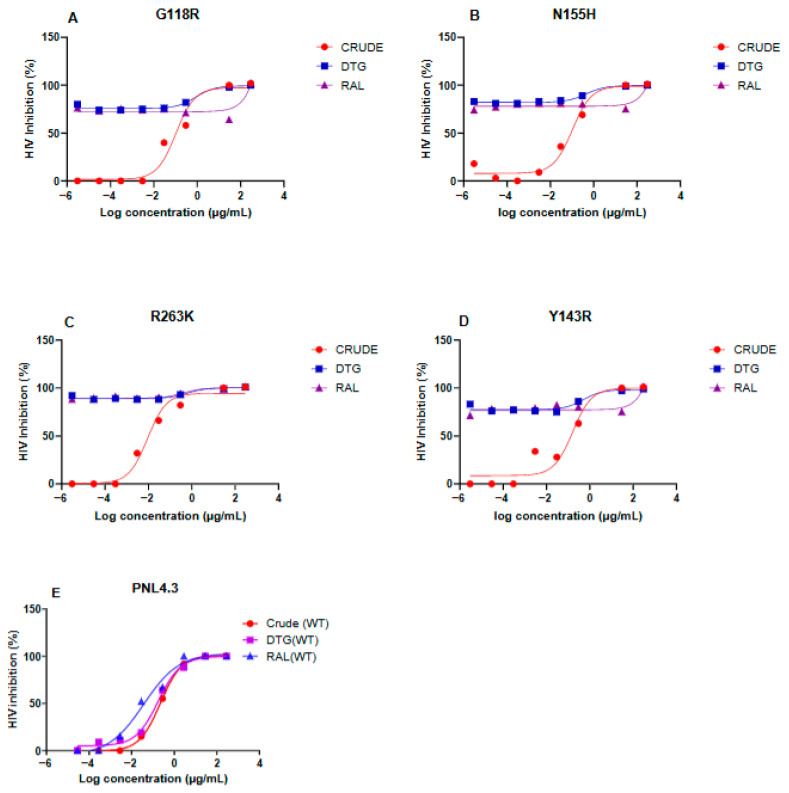
**Inhibitory efficacy of *A. alternata* PO4PR2 crude extract against various HIV-1 integrase (IN) drug-resistant mutant strains and the wild-type control**. (**A**) G118R, (**B**) N155H, (**C**) R263K, (**D**) Y143R, (**E**) pNL4.3. Data are represented as the mean ± standard deviation (SD) from three independent experiments. Statistical significance compared to the wild-type control is indicated in panel (**E**), where *p* < 0.05.

The antiviral efficacy of the *A. alternata* PO4PR2 crude extract was evaluated alongside clinical drug controls (Dolutegravir and Raltegravir) against the site-directed mutants G118R, N155H, R263K, and Y143R. Dose–response curves (Figure 4A–E) demonstrated that viral replication for all strains was completedly inhibited at a concentration of 300 µg/mL. The crude extract exhibited high potency, with IC_50_ values for all mutants falling well below the 10 μg/mL for benchmark for natural producty activity.

**Figure 5:**
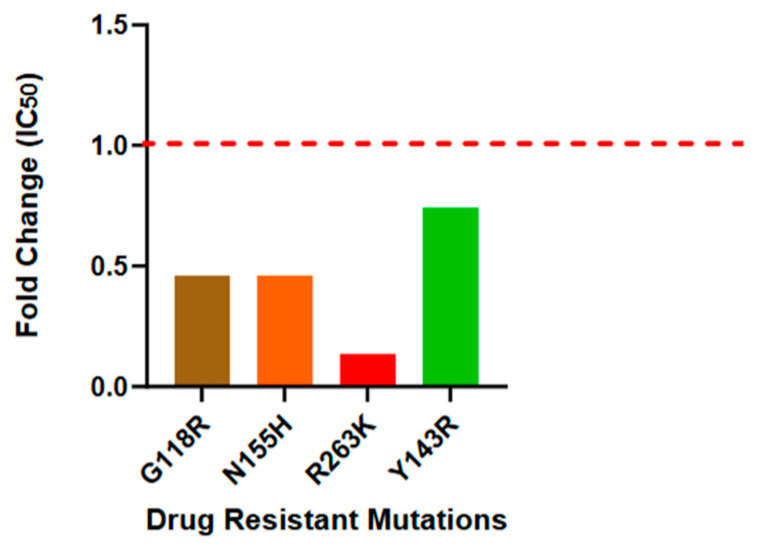
**Fold change in IC_50_ values of the *A. alternata* PO4PR2 crude extract against HIV-1 INSTI-resistant mutants.** The bars represent the fold changes in susceptibility of the G118R, N155H, R263K, and Y143R site-directed mutants relative to the wild-type pNL4.3 reference strain (indicated by the dashed red lines at y = 1.0). Fold-change values were calculated as the ratio of the mutant IC50 to the wild-type Ic50. While G118R, N155H, and Y143R exhibited a modest reduction in susceptibility (fold change ≈ 2.0), the R263K variant showed unique hypersensitivity to the extract (fold change ≤1.0), suggesting a mutation-tolerant inhibitory mechanism.

The suceptibility profile for each mutant was further quantified by calculating the fold change in IC_50_ relative to the wild-type pNL4.3 reference (Table 3; Figure 5). Fold-change values greater than 1, observed for G118R (1.43), N155H (1.41), and Y143R (2.26), indicated a modest reduction in suceptibility compared to the wild-type. By contrast, the R263K variant exhibited a fold change of 0.41, indicating significant hypersensitivity to the crude extract. This suggests that *A. alternata* PO4PR2 extract utilizes a mechanism of action that remains highly effective, or even enhanced, in the presence of the R263K substitution.

#### 3.2.3. Comparative Analysis of Efficacy and Selectivity Index (SI)

To evaluate the therapeutic potential of the *A. alternata* PO4PR2 crude extract, the Selectivity Index (SI) was calculated as the ratio of CC_50_/CC_50_ [38]. In this study, PO4PR2 extract exhibited an exceptional safety profile, with SI values ranging from 1846 to over 10,000 across the mutant panel (Table 4). Notably, the extract’s SI values were frequently higher than those observed for the Dolutegravir (DTG) control, primarily due to the extract’s high potency against resistant variants. The susceptibility of each mutant was further quantified by expressing IC_50_ values as a fold change relative to the pNL4.3 reference strain (Figure 5). While G118R, N155H, and Y143R showed a modest reduction in susceptibility (fold change ≈ 2.0), the absolute IC_50_ values remained in the sub-microgram range. By contrast, the R263K variant demonstrated increased susceptibility to the extract (fold-change ≤ 1.0), corroborating the “mutation-tolerant” profile of the fungal metabolites.

#### 3.2.4. *In Vitro* Inhibition of HIV-1 Integrase Strand-Transfer Activity

**Figure 6:**
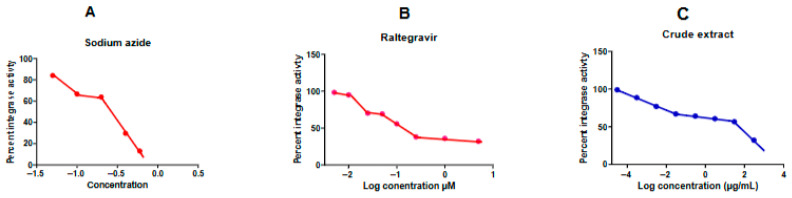
Biochemical inhibition of HIV-1 integrase strand-transfer activity. Dose–response curves showing the inhibitory effects of (**A**) sodium azide, (**B**) Raltegravir (positive control), and (**C**) *A. alternata* PO4PR2 crude extract on recombinant integrase. Each data point represents the mean ± SD of three independent experiments. The extracts (**C**) demonstrate potent, dose-dependent inhibition (IC50 = 2.85 ug/mL), supporting a mechanism of action targeting the strand-transfer reaction.

The inhibitory potential of the *A. alternata* P04PR2 crude extract against recombinant HIV-1 integrase was evaluated using a strand transfer-specific assay. As shown in Figure 6C, the extract demonstrated dose-dependent inhibition of enzymatic activity with an IC_50_ of 2.85 µg/mL. By comparison, the clinically approved INSTI Raltegravir exhibited the highest a superior potency of IC_50_ of 0.03477 µg/mL (Figure 6B), while sodium azide, used here as a non-specific inhibitory control, yielded an IC_50_ of 0.7654 µg/mL (Figure 6A). Although the purified reference drug (Raltegravir) showed higher absolute potency, the low-microgram IC_50_ of the *A. alternata* crude extract is highly significant for a non-purified natural product. This suggests that the extract contains potent secondary metabolites capable of specifically disrupting the integrase–DNA complex. These results corroborated the antiviral activity observed in the TZM-bl reporter assays and provide biochemical evidence that the extract’s mechanism involves the direct blockade of the HIV-1 strand-transfer step. Further bioassay-guided fractionation is warranted to isolate the specific constituents responsible for this high-level enzymatic inhibition.

#### 3.2.5. Quantification of Proviral Integration via *Alu-Gag* Nested PCR

To evaluate the effect of the *A. alternata* PO4PR2 crude extract on proviral integration, infected PBMCs from 20 HIV-1 negative donors were analyzed using the two-step *Alu-gag* nested PCR assay. Following infection with the site-directed mutants (G118R, N155H, R263K, and Y143R), cells were treated with the crude extract, Raltegravir (RAL), or Dolutegravir (DTG), with the results normalized to human *GAPDH*.

**Figure 7:**
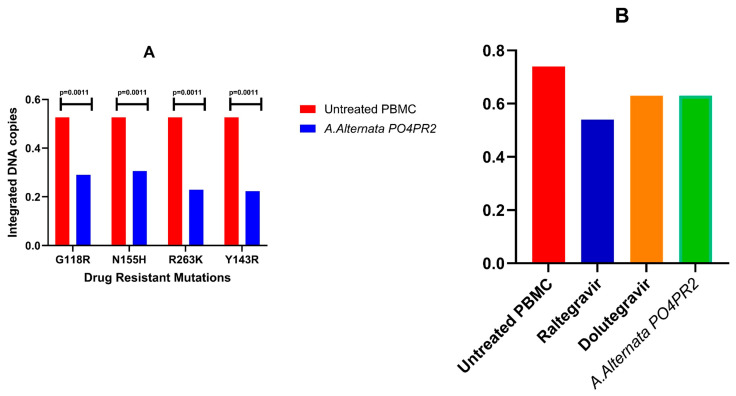
**Inhibition of HIV-1 proviral integration by *A. alternata* PO4PR2 crude extract measured using *Alu-gag* nested PCR**. (**A**) Quantification of integrated HIV-1 DNA copies across four INSTI-resistant pathways (G118R, N155H, R263K, and Y143R) following treatment with *A. alternata* PO4PR2 extract. (**B**) Comparative inhibitory efficacy of the crude extract against clinical INSTIs (Raltegravir and Dolutegravir). All data were normalized to GAPDH and represent the mean ± SD of 20 donors. Statistical significance was determined at *p* = 0.0011 relative to untreated infected controls.

Treatment with the *A. alternata* extract resulted in a significant reduction in integrated HIV-1 DNA copies across all tested mutants (Figure 7A). In untreated control cells, integration levels ranged from 0.50 to 0.55 copies/cells. Upon treatment with PO4PR2 extract, these levels were reduced to 0.23–0.30 copies/cell (*p* = 0.0011). Notably, the inhibitory effect of the crude extract was comparable to that of Dolutegrvir (approximately 0.20 copies/cell), while Raltegravir showed the most pronounced reduction (Figure 7B).

These findings demonstrate that the *A. alternata* PO4PR2 crude extract specifically targets the integration step of the viral life cycle, maintaining efficacy even against established INSTI-resistant variants. The ability of a crude fungal extract to approach the potency of licensed antiretroviral drugs like DTG highlights the presence of highly active metabolites warranting further purification and mechanistic optimization.

#### 3.2.6. Molecular Docking Analysis

##### Binding Affinity Analysis

**Figure 8:**
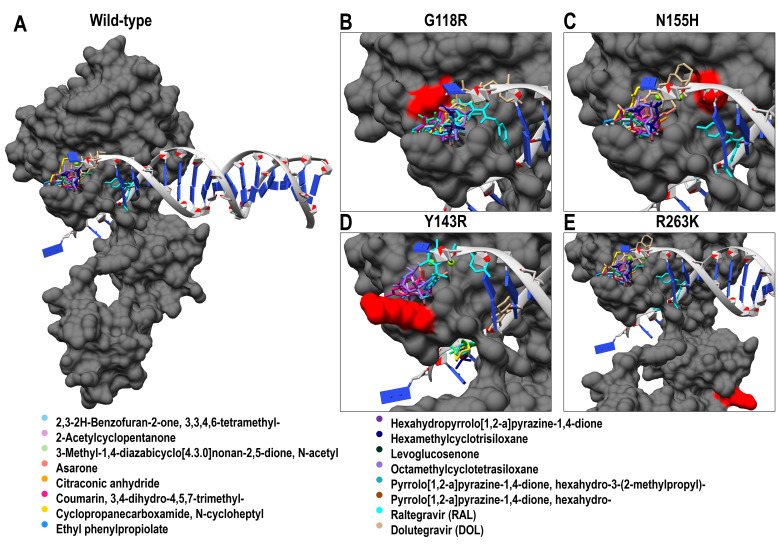
**Molecular docking poses of lead *A. alternata* PO4PR2 metabolites within the HIV-1 integrase catalytic pocket.** (**A**–**E**) Comparative binding orientations of 3,4-dihydro-4,5,7-trimethyl-coumarin and 3,3,4,6-tetramethyl-2,3-2H-benzofuran-2-one within the active sites of wild-type and mutant (G118R, N155H, R263K, and Y143R) integrase enzymes. The metabolites (shown in stick representation) occupy the same catalytic space as clinical INSTIs, interacting with key conserved residues and coordinating with the active site magnesium (Mg) co-factors. Hydrogen bonds and hydrophobic interactions are indicated to illustrate the stability of the binding complex across resistant variants. Images were generated and visualized using PyMoL version 3.1 or Discovery Studio.

Molecular docking simulations were performed against the catalytic core domains of HIV-1 integrase, including the wild-type and four clinically relevant resistant mutants (G118R, N155H, Y143R, and R263K). The binding poses of the 14 metabolites were visualized and compared with those of the reference inhibitors, Raltegravir and Dolutegravir, across both wild-type and mutant integrase structures (Figure 8). All metabolites occupied the catalytic pocket, aligning with the binding orientations of the reference drugs and indicating potential engagement with key residues essential for strand-transfer inhibition.

**Figure 9:**
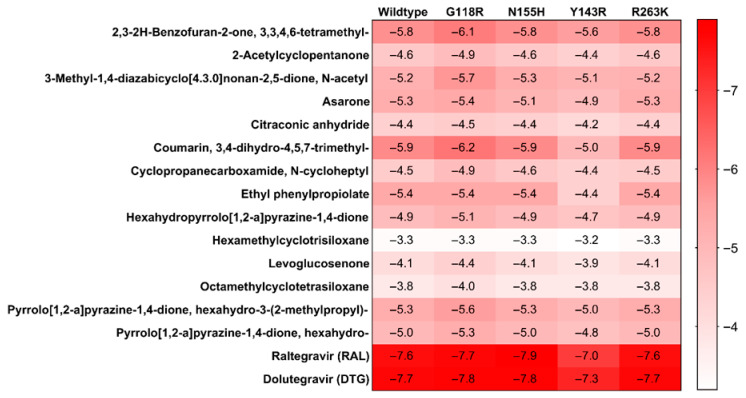
**Molecular docking scores (kcal/mol) of *A. alternata* PO4PR2 metabolites against HIV-1 IN and HIV-1 mutant strain.** The heatmap/bar chart displays the predicted binding affinities (kcal/mol) for the 14 identified secondary metabolites. More negative values indicate stronger ligand–protein interactions. 3,4-dihydro-4,5,7-trimethyl-coumarin and 3,3,4,6-tetramethyl-2,3-2H-benzofuran-2-one emerged as the top-scoring leads, maintaining consistent binding stability (−5.6 to −6.8 kcal/mol) across the wild-type (pNL4.3) and the resistant variants (G118R, N155H, R263K, and Y143R). These scores suggest a mutation-tolerant binding profile when compared to the reference inhibitors Raltegravir and Dolutegravir.

More negative docking scores correspond to higher predicted binding affinities (Figure 9). Among the screened compounds, 3,4-dihydro-4,5,7-trimethyl-coumarin exhibited the most favorable binding across all IN variants, with scores ranging from −5.9 to −6.2 kcal/mol. This was followed closely by 3,3,4,6-tetramethyl-2,3-2H-benzofuran-2-one (−5.6 to −6.1 kcal/mol) and hexahydro-3-(2-methylpropyl)-pyrrolo[1,2-a] pyrazine-1,4-dione (−5.0 to −5.6 kcal/mol). These results suggest that the metabolites bind within the same catalytic pocket but with moderate predicted affinity, consistent with their activity as part of a crude extract.

By contrast, compounds such as hexamethylcyclotrisiloxane and octamethylcyclotetrasiloxane displayed the weakest affinities (−3.2 to −3.8 kcal/mol), suggesting limited potential for integrase inhibition. While the control drugs, Raltegravir and Dolutegravir, demonstrated superior docking scores (−7.0 to −7.9 kcal/mol), the consistent performance of the coumarin and benzofuranone scaffolds across resistant variants provide a molecular basis for the mutation-tolerant profiles observed in our in vitro assays.

##### Protein–Ligand Interaction Analysis

**Figure 10:**
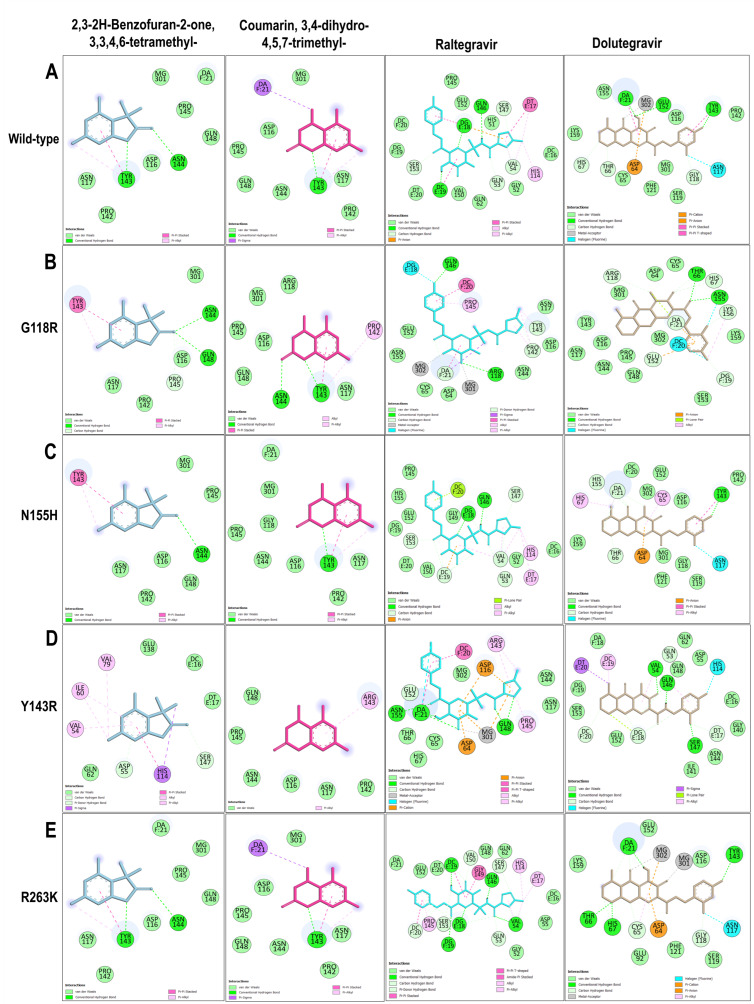
**Interaction maps of *A. alternata* metabolites within the active site of wild-type and mutant IN–DNA–Mg^2+^ complexes**. (**A**) Wild-type, (**B**) G118R, (**C**) N155H, (**D**) Y143R, (**E**) R263K.

Comparative protein–ligand interaction profiling was performed for the *A. alternata* metabolites 3,3,4,6-tetramethyl-2,3-2H-benzofuran-2-one and 3,4-dihydro-4,5,7-trimethyl-coumarin against wild-type and four HIV-1 IN variants (G118R, N155H, Y143R, and R263K). Reference inhibitors Dolutegravir (DTG) and Raltegravir (RAL) were included to delineate the impact of resistance-associated mutations on ligand recognition and metal coordination within the IN–DNA–Mg^2+^ catalytic complex (Figure 10). In the wild-type IN–DNA landscape, both *A. alternata* PO4PR2 metabolites were stably anchored within the catalytic pocket at the IN–DNA interface. 3,3,4,6-Tetramethyl-2,3-2H-benzofuran-2-one established hydrogen bonds with ASN144 and TYR143, supplemented by π–π stacking and π–alkyl interactions with TYR143. Van der Waals interactions involving ASP116, ASN117, PRO142, PRO145, GLN148, and the Mg^2+^ ion (Mg301) further stabilized the complex. Likewise, 3,4-dihydro-4,5,7-trimethyl-coumarin formed hydrogen bonds, π–π stacking, and π–alkyl interactions with TYR143, alongside a π–sigma contact with the DNA base DA F:21. The persistence of π–π stacking with TYR143 across both ligands underscores the central role of this residue in ligand anchoring at the active site. By contrast, the reference inhibitors exhibited distinct binding characteristics. Raltegravir formed hydrogen bonds with GLN53, GLN146, SER147, and SER153, along with π–alkyl interactions involving VAL54 and HIS114, but lacked direct metal coordination. Additional hydrogen bonding, hydrophobic contacts, and van der Waals interactions with DNA base pairs further stabilized the binding within the IN–DNA complex. However, Dolutegravir demonstrated stronger metal–acceptor coordination with Mg^2+^ (Mg302) and formed hydrogen bonds with THR66, HIS67, GLY118, TYR143, and GLU152, complemented by π–anion, π–π stacking, and halogen (fluorine) interactions (Figure 10A). These extensive polar and coordination contacts reflect the deeper and more stable engagement of Dolutegravir within the catalytic triad compared to that of Raltegravir.

Upon introduction of the G118R mutation, both metabolites retained their key aromatic stacking interactions with TYR143, indicating structural tolerance to perturbations near the catalytic core. 3,3,4,6-Tetramethyl-2,3-2H-benzofuran-2-one gained additional hydrogen bonds with PRO145 and GLN148, while 3,4-dihydro-4,5,7-trimethyl-coumarin maintained TYR143 binding and formed new hydrogen bonds with ASN144 alongside alkyl contacts with PRO142. These compensatory interactions suggest adaptive stabilization, despite the steric influence of ARG118. By comparison, Raltegravir exhibited extended coordination with both Mg^2+^ ions, forming hydrogen bonds with ARG118, TYR143, PRO142, and GLN146, as well as π–π stacking with DC F:20, whereas Dolutegravir preserved its canonical halogen, hydrogen bonding (THR66, HIS67, GLU152), and π–anion contacts (Figure 10B). For the N155H variant, both *A. alternata* PO4PR2 metabolites maintained conserved hydrogen bonding with ASN144 (3,3,4,6-tetramethyl-2,3-2H-benzofuran-2-one) or TYR143 (3,4-dihydro-4,5,7-trimethyl-coumarin) and retained π–π stacking with TYR143. Their interaction networks were largely unaltered compared to those of the wild-type, indicating minimal disruption by the N155H substitution. Correspondingly, Raltegravir and Dolutegravir sustained strong coordination with DNA bases and Mg^2+^ ions, confirming that this mutation exerts limited influence on ligand positioning at the catalytic triad (Figure 10C). In the Y143R variant, a marked conformational reorganization occurred around the active site loop. 3,3,4,6-Tetramethyl-2,3-2H-benzofuran-2-one shifted its π–π stacking from TYR143 to HIS114, forming hydrogen bonds with ASP55 and SER147 and hydrophobic contacts with ILE60 and VAL79. Conversely, 3,4-dihydro-4,5,7-trimethyl-coumarin displayed weakened binding, restricted to π–alkyl interactions with ARG143 and van der Waals interactions within the catalytic loop. By comparison, Raltegravir maintained robust hydrogen bonding and metal coordination, whereas Dolutegravir formed multiple stabilizing contacts with DNA bases (Figure 10D). For the R263K variant, both metabolites recapitulated the wild-type interaction profiles, maintaining hydrogen bonds and π–π stacking with TYR143 and van der Waals interactions with PRO142, PRO145, and GLN148. The persistence of TYR143-mediated interactions signifies a strong conformational conservation around the DNA-binding groove. Raltegravir retained its DNA base contacts, whereas Dolutegravir continued to exhibit robust Mg^2+^ coordination (Figure 10E).

## 4. Discussion

*Alternaria* species are prolific producers of secondary metabolites, including polyketides, terpenoids, and alkaloids, many of which exhibit significant biological activities. *A. alternata* PO4PR2 crude extracts isolated from the roots, stems, and leaves of *Sclerocarya birrea* and *Hypoxis* plants were found to inhibit HIV-1 replication at various stages [20]. While previous studies identified the anti-HIV potential of *A. alternata* PO4PR2 extract [21,22], the precise mechanisms and efficacy against drug-resistant variants remained speculative. This study is the first to functionally validate the extract’s activity against a specific panel of site-directed mutants (G118R, N155H, R263K, and Y143R). Our findings demonstrate that the *A. alternata* PO4PR2 extract maintains potent inhibitory activity across these pathways, which represent the most clinically challenging INSTI-resistance profiles currently circulating in Southern Africa.

The anti-HIV-1 efficacy of the *A. alternata* PO4PR2 crude extract was evaluated against a panel of INSTI mutants (G118R, N155H, R263K, and Y143R) using a luciferase-based reporter assay. Across all variants, the extract maintained potent inhibition with geometric mean IC_50_ values consistently below 10 µg/mL (Table 3), a recognized benchmark for high antiviral activity in natural products [39,40]. While the G118R, N155H, and Y143R pathways exhibited a degree of reduced susceptibility (fold change >1) relative to pNL4.3 reference strains, the absolute inhibitory concentrations remained within a therapeutic range (Figure 5). Notably, the R263K variant displayed unique hypersensitivity to the extract (fold change <1). This contrasts with its known resistance to second-generation INSTIs like Dolutegravir (DTG) [22] and suggests that the extract’s metabolites may utilize a binding mechanism that is not compromised by the structural shifts associated with R263K substitution.

In the present study, the site-directed mutants (G118R, N155H, R263K, and Y143R) exhibited diminished replication capacity compared to the wild-type pNL4.3 reference. This observed reduction in infectivity aligns with established reports describing the fitness costs associated with integrase strand transfer inhibitor (INSTI) resistance [41]. Specifically, the R263K mutation is known to reduce HIV-1 replication fitness and subtly alter the structural configuration of the integrase catalytic pocket, which specifically hinders the binding orientation of Dolutegravir (DTG) [41,42]. Similarly, the G118R mutation structurally compromises wild-type integrase–DNA complexes, leading to impaired viral fitness [43,44]. These fitness costs represent a common trade-off where the virus sacrifices replicative efficiency to survive drug selective pressure. We hypothesize that the enhanced susceptibility to these mutants to the *A. alternata* crude extract is due to the structural diversity of its secondary metabolites. Unlike the rigid scaffold of second-generation INSTIs, the extract contains multiple coumarin and phenolic derivatives that may utilize alternative hydrogen bonding networks or hydrophobic interactions that are not sterically hindered by the lysine (K) substitution [22]. This suggests a mutation-tolerant binding mechanism where the extract components target residues that remain conserved or accessible even in the R263K variant.

Our findings provide the first functional evidence that *A. alternata* PO4PR2 metabolites maintain efficacy against the most challenging INSTI-resistance profiles circulating in Southern Africa. The mutations selected, specifically G118R and R263K, are drivers of virological failure from second-generation inhibitors [45,46,47]. The fact that our extract retained activity against G118R, a mutation that causes significant structural incompatibility for current clinical inhibitors, suggests a robust and potentially novel mechanism of action. This mechanism was further elucidated through enzymatic and proviral DNA assays. The HIV-1 integrase inhibition assay confirmed that the extract targets the strand-transfer step, mirroring the pharmacological profile of clinical INSTI [48,49,50,51,52]. This was corroborated by *Alu-gag* PCR analysis, which showed a significant, dose-dependent reduction in integrated proviruses (Figure 6). Such dual-track validation, combining cell-free enzymatic data with cellular integration assays, provides convergent evidence that the extract disrupts the core machinery of viral integration.

Molecular docking analysis identified 3,4-dihydro-4,5,7-trimethyl-coumarin and 3,3,4,6-tetramethyl-2,3-2H-benzofuran-2-one as the primary bioactive leads (Figure 7). With favorable binding affinities (−6.2 to −6.8 kcal/mol) across both wild-type and mutant strains, these compounds appear structurally optimized to occupy the catalytic pocket and disrupt the integration stage. In particular, the strong predicted interaction and stable binding of the benzofuranone derivative suggests that it may be a primary mediator of the extract’s inhibitory profile. These metabolites formed stable interaction networks with conserved residues, such as TYR143 and ASN144, while simultaneously coordinating with Mg^2+^ co-factors [22]. This binding orientation, comparable to that exploited by clinical INSTIs, provides a molecular rationale for our in vitro findings and strongly supports the role of these metabolites as strand-transfer inhibitors.

The identification of these scaffolds aligns with previous GC-MS characterization of *A. alternata* PO4PR2 extract, which highlighted a diverse chemical profile, including pyrrolo[1,2-a] pyrazine-1,4-dione, 3,4-dihydro-4,5,7-trimethyl-coumarin, and 4,5,7-trimethyl-2-chromanone [20]. Notably, our findings corroborate recent docking studies where similar scaffolds demonstrated stability against the T66K and S230R variants, suggesting a consistent, mutation-tolerant mechanism of action. By integrating computational docking, enzymatic assays, and proviral integration studies, this research provides robust evidence that *A. alternata* PO4PR2 metabolites function as potent strand-transfer inhibitors. Coupled with a high Selectivity Index (SI > 1000), these results reinforce the potential of fungal-derived scaffolds as safe, next-generation antivirals capable of overcoming current barriers in HIV-1 drug resistance.

### Limitations of the Study

While these results are promising, several limitations should be noted. First, the use of crude extracts means that the specific bioactive metabolites responsible for the observed inhibition and any potential synergistic interactions between them have not yet been isolated or quantified. Second, although our in vitro assays are well-validated, they do not account for the complex pharmacokinetics or immune responses present in vivo. Furthermore, our focus was restricted to the pNL4.3 backbone and four specific mutations. Future research should expand this to include HIV-1 subtype C clinical isolates, which are predominant in Southern Africa. Finally, while we demonstrated potent inhibition, further mechanistic studies and drug-likeness evaluations, including metabolic stability and membrane permeability, are essential for determining the clinical viability of these fungal-derived scaffolds.

## 5. Conclusions

This study provides compelling evidence that *A. alternata* PO4PR2 serves as a prolific source of metabolites capable of disrupting the HIV-1 life cycle. Mechanistically, our findings demonstrate that the *A. alternata* crude extract functions as a strand-transfer inhibitor, effectively blocking the covalent insertion of proviral DNA into the host genome. The identification of lead integrase-binding compounds, paired with the marked reduction in integrated DNA observed via *Alu-gag* PCR, highlights the potential of these fungal chemotypes for overcoming established resistance barriers. These results provide a strong rationale for the further isolation and optimization of these scaffolds as next-generation antiretroviral therapeutics.

## Figures and Tables

**Table 1:** Resistance scores of selected HIV-1 integrase mutations.

DRM	Bictergravir (BIC)	Cabotegravir (CAB)	Dolutegravir (DTG)	Elvitegravir (EVG)	Raltegravir (RAL)
**G118R**	45	60	60	60	60
**N155H**	10	30	10	60	60
**Y143R**	0	10	0	10	60
**Q148R**	25	60	25	60	60
**T66A**	0	0	0	60	15
**E138K**	10	15	10	15	15

Resistance levels are based on Stanford HIVDB penalty scores: 0–9 (susceptible), 15–29 (low-level), 30–59 (intermediate), and ≥60 (high-level resistance).

**Table 2:** Mutagenic primers for HIV-1 integrase variants.

Primer Name	Sequence (5′–3′)	Targeted Mutation
N155H_FWD	AGAATCTATGC**A**TAAAGAATTAAAGAAAATTATAG	AAC→ CAT
N155H_REV	ACTACTCCTTGACTTTGG	
Y143R_FWD	TGGAATTCC**C**GCAATCCCCAAAG	TAC → CGC
Y143R_REV	AATTCCTGCTTGATTCC	
G118R_FWD	TACTGACAAT**C**GCAGCAATTTCAC	GGA → CGC
G118R_REV	TGTATTGTTTTTACTGGCC	
Q148R_FWD	TCCCCAAAGTCG**C**GGAGTAGTAG	CAG →CGC
Q148R_REV	TTGTAGGGAATTCCAAATTC	
Q148K_FWD	TCCCCAAAGT**AAA**GGAGTAGTAG	CAG→AAA
Q148K_REV	TTGTAGGGAATTCCAAATTC	
R263K_FWD	AAAGATTGT**AAA**TACTTACAAGGAG	AGA →AAA
R263K_REV	CATGTTCTAATCCTCATCCT	

**Table 3:** IC_50_ values and fold change in susceptibility for HIV-1 integrase mutants.

HIV-1 Strain	IC_50_ (µg/mL) ± SD	Fold-Change	Susceptibility Profile
pNL4.3 (WT)	0.073 ± 0.012	1.00	Reference
G118R	0.104 ± 0.023	1.43	Reduced
N155H	0.103 ± 0.025	1.41	Reduced
R263K	0.030 ± 0.009	0.41	Hypersensitive
Y143R	0.165 ± 0.027	2.26	Reduced

Note: Fold change = (Mutant IC_50_/pNL4.3 IC_50_) values are unified to three decimal places.

**Table 4:** IC_50_ values and Selectivity Index (SI) of PO4PR2 extract and Dolutegravir.

HIV-1 Strain	IC_50_ PO4PR2 (µg/mL) ± SD	SI (PO4PR2)	IC_50_ DTG (µg/mL) ± SD	SI (DTG)
pNL4.3 (WT)	0.073 ± 0.015	4110	0.177 ± 0.002	1695
G118R	0.104 ± 0.023	2885	0.855 ± 0.003	351
N155H	0.103 ± 0.025	2913	0.443 ± 0.003	677
R263K	0.030 ± 0.009	10,000	0.619 ± 0.004	485
Y143R	0.165 ± 0.027	1818	0.503 ± 0.003	596

Abbreviations: SD, standard deviation; DTG, Dolutegravir; SI, Selectivity Index (CC_50_/IC_50_). IC_50_ values represent the mean of three independent experiments. All IC_50_ values are reported to three decimal places.

## Data Availability

Data available within the article.
